# Integrating speech biomarkers and large language models for adolescent suicide risk detection with mobile application for real-world evaluation

**DOI:** 10.1016/j.xcrm.2026.102823

**Published:** 2026-06-16

**Authors:** Chang Lei, Ziyun Cui, Yinan Duan, Zhijun Wu, Diyang Qu, Wen Wu, Zeming Zhang, John S. Ji, Bowen Zhou, Ji Wu, Chao Zhang, Runsen Chen

**Affiliations:** 1 Vanke School of Public Health, Tsinghua University, Beijing, China; 2 Department of Electronic Engineering, Tsinghua University, Beijing, China; 3 Shanghai Artificial Intelligence Laboratory, Shanghai, China

**Keywords:** adolescent suicide risk, speech biomarkers, large language models, mobile application, real-world evaluation

## Abstract

Adolescent suicide is a significant public health issue, highlighting the need for efficient methods to detect suicide risk. Here, we develop and validate a speech-based suicide risk detection framework grounded in large language models (LLMs). Two independent cohorts of adolescents aged 10–18 years are analyzed: a development cohort (*n* = 1,223), with voice recordings collected in structured interview settings for model training and internal evaluation, and an external validation cohort (*n* = 460), collected through a mobile application to assess feasibility in naturalistic settings. An integrated model combining a speech encoder and an LLMs-based text-processing branch achieves its best performance on the *self-introduction* task. The model yields an accuracy of 0.808 and a macro-F1 score of 0.807 for suicide risk detection and remains effective under naturalistic mobile assessment. These findings support integrating LLMs with speech-derived markers for scalable adolescent suicide risk detection.

## Highlights

• Large language model-integrated speech analysis detects adolescent suicide risk
• Ten speech tasks enable comprehensive evaluation of detection performance
• Our framework detects adolescent suicide risk with 0.808 accuracy
• External validation using a mobile application supports real-world applicability

## Graphical abstract

**Figure:**
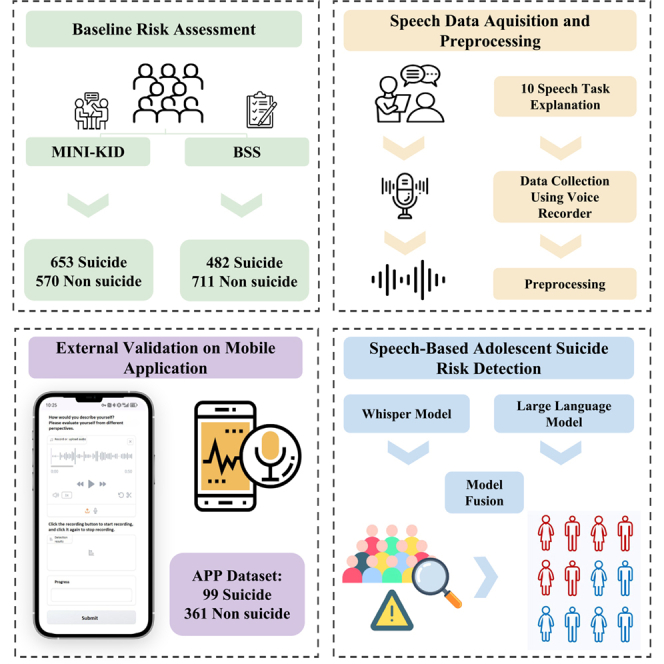

## Teaser

Lei et al. develop a speech-based framework integrating a speech encoder and large language models to detect adolescent suicide risk. Across ten speech tasks, the *self-introduction* task demonstrates the strongest performance. The framework outperforms conventional baselines and unimodal models and remains effective in mobile application-based external validation.

## Introduction

Suicide is a critical public health issue and ranks as one of the leading causes of death among adolescents worldwide.^1^ Although early suicide risk detection is essential for effective prevention and intervention, studies indicate that current predictive capabilities remain only marginally better than chance and have shown little improvement over time.^2^ In line with these findings, traditional statistical methods and risk prediction models have yet to yield the desired level of clinical effectiveness.^3^ Furthermore, self-report screening tools commonly used in schools still face significant challenges, including issues of discrimination and barriers to accurate reporting,^4^ while clinical interviews, though more accurate, are highly resource-intensive. These circumstances underscore the need for innovative approaches that may offer more accessible and less biased ways to improve suicide risk detection.

Speech, with its rich paralinguistic and linguistic information, offers a cost-effective tool for identifying and predicting suicide risk.^5^ Previous studies have shown the utility of speech as a marker for distinguishing mental states and psychiatric disorders,^6^ and similar associations have been reported in research focused specifically on suicide risk.^5^^,^^7^ Machine learning and signal processing approaches applied to speech features have shown promise^8^^,^^9^^,^^10^; however, traditional feature extraction methods remain limited in their transferability across languages and domains. Their focus on surface-level characteristics such as pitch and tone may also make it more difficult to capture the deeper emotional and cognitive signals that are important for identifying suicide risk.^11^ In addition, they offer only limited capacity to process speech and text jointly, which further constrains their ability to reflect the complex expressive patterns associated with suicide risk.^12^

In recent years, foundation models such as large language models (LLMs),^13^^,^^14^ pre-trained on vast and heterogeneous corpora, capture complex contextual and semantic information beyond the capabilities of earlier models^15^^,^^16^ and have demonstrated utility in mental health applications.^17^^,^^18^^,^^19^ Multimodal LLMs integrating speech, text, and visual information have further broadened representational scope.^20^^,^^21^ Architectures that combine speech encoders with LLM-based decoders, along with unified fusion frameworks, enable models to learn jointly from speech and text. This design allows the integration of prosodic, affective, and linguistic cues in ways that are not readily attainable with traditional feature extraction methods.^22^^,^^23^ Despite these developments, the use of multimodal LLMs for speech-based suicide risk detection remains underexplored, with most studies relying on isolated or narrowly defined tasks.^5^^,^^8^^,^^9^^,^^10^ There is a clear need for task paradigms that elicit a broad range of linguistic, cognitive, and emotional responses, as these can support a more comprehensive assessment of factors linked to suicide risk. Such paradigms also create the conditions necessary for speech-text multimodal LLMs, which depend on jointly informative inputs, to more effectively capture the communicative and affective patterns associated with suicide risk.

In our previous conference paper,^24^ we introduced a framework for suicide risk detection using spontaneous speech processed through a foundation model. This initial study employed a standardized *self-introduction* speech task to assess suicide risk in school-aged adolescents, using diagnostic labels from the Mini International Neuropsychiatric Interview for Children and Adolescents (MINI-KID). Although the model achieved effective predictive performance, it was based on a single speech task and developed within a controlled recording environment, potentially limiting its generalizability to real-world applications.

Therefore, the present study introduces methodological and design enhancements alongside expanded validation efforts. First, we substantially broadened the speech task evaluation process by incorporating ten distinct tasks, spanning both structured and unstructured formats, to ensure a more comprehensive assessment of suicide risk indicators. Second, in addition to the MINI-KID diagnostic interview, we incorporated the Beck Scale for Suicide Ideation (BSS) as a complementary assessment of current suicidal ideation, which represents an early and clinically consequential indicator of vulnerability. Third, to enhance ecological validity, we developed and deployed a mobile application to enable real-time speech data acquisition beyond laboratory constraints. Concurrently, we fine-tuned and validated our model using mobile application-based speech data, demonstrating its adaptability in real-world settings. Finally, to assess model generalizability, we conducted external validation across multiple schools, further strengthening the evidence for its practical applicability. These enhancements collectively represent a substantial advancement, improving key methodological components and advancing the feasibility of speech-based suicide risk detection in real-world contexts.

## Results

### Demographic statistics

The first stage of data collection involved a total of 1,223 adolescents from 47 schools, of which 653 (53.4%) were identified as being at current suicide risk based on the MINI-KID interview, with 482 (40.4%) self-reporting current suicidal ideation using the BSS scale. During the mobile application phase, a total of 460 adolescents across 14 schools completed the assessment, of which 99 (21.5%) were identified as being at current suicide risk based on the MINI-KID interview. Details of the demographic characteristics are provided in Table 1.

**Table 1:** Demographic characteristics of participants in the voice recorder- and mobile application-based speech data

MINI-KID label (voice recorder-based speech data)						
	Yes (653, 53.4%)			No (570, 46.6%)		
Gender	male, female 186 (28.5%), 467 (71.5%)			male, female 246 (43.2%), 324 (56.8%)		
Age	10 to 12 128 (19.6%)	13 to 15 309 (47.3%)	16 to 18 216 (33.1%)	10 to 12 149 (26.1%)	13 to 15 256 (44.9%)	16 to 18 165 (29.0%)

BSS label (voice recorder-based speech data)						
	Yes (482, 40.4%)			No (711, 59.6%)		
Gender	male, female 121 (25.1%), 361 (74.9%)			male, female 299 (42.1%), 412 (57.9%)		
Age	10 to 12 84 (17.4%)	13 to 15 255 (52.9%)	16 to 18 143 (29.7%)	10 to 12 180 (25.3%)	13 to 15 295 (41.5%)	16 to 18 236 (33.2%)

MINI-KID label (mobile application-based speech data)						
	Yes (99, 21.5%)			No (361, 78.5%)		
Gender	male, female 24 (24.2%), 75 (75.8%)			male, female 154 (42.7%), 207 (57.3%)		
Age	10 to 12 28 (28.3%)	13 to 15 54 (54.5%)	16 to 18 17 (17.2%)	10 to 12 218 (60.4%)	13 to 15 108 (29.9%)	16 to 18 35 (9.7%)

Statistics for the speech data at each stage are listed. MINI-KID: Mini International Neuropsychiatric Interview for Children and Adolescents; BSS, Beck Scale for Suicide Ideation.

### Results from voice recorder-based speech data in structured interview settings

#### Model results derived from MINI-KID label

**Figure 1:**
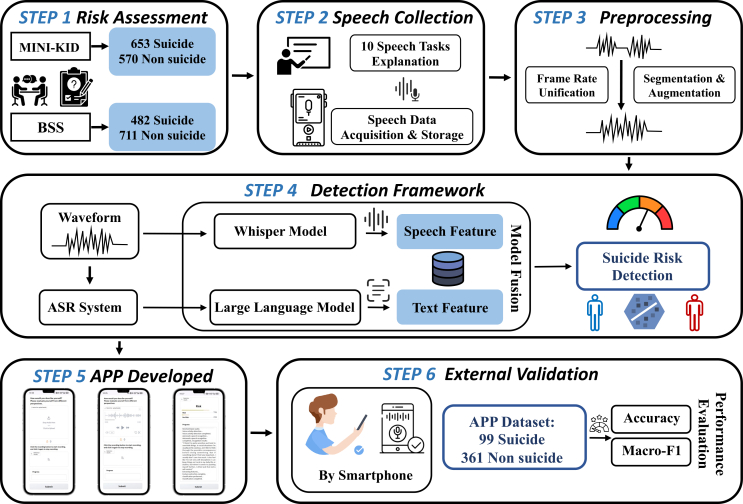
Workflow of the study design and suicide risk detection framework Suicide risk was assessed via the MINI-KID interview, with suicidal ideation characterized using the BSS self-report, and speech data collected across ten tasks. The collected speech data were preprocessed and fed into our detection framework, comprising a speech branch and a text branch. Features from both modalities were fused to perform suicide risk detection. The constructed framework was subsequently validated on mobile application-based speech data in real-world settings.

**Figure 2:**
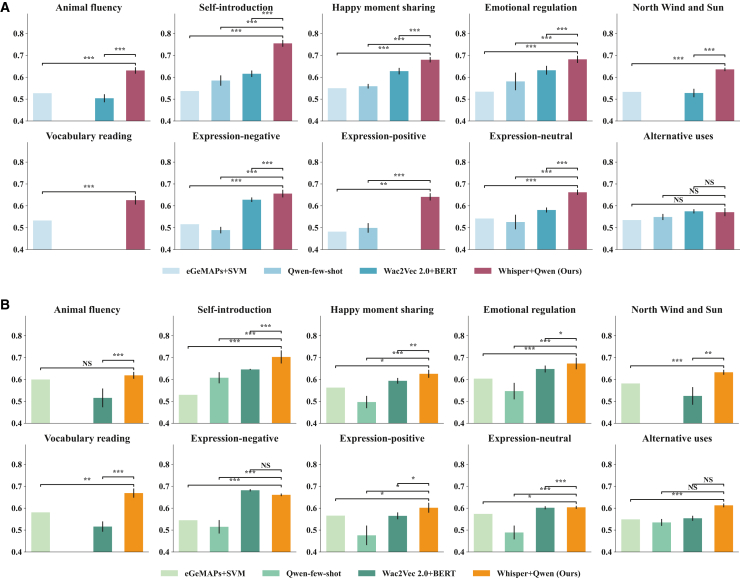
Accuracy comparison across methods for MINI-KID and BSS labels (A) Accuracy comparison across methods on ten speech tasks using the MINI-KID label. (B) Accuracy comparison across methods on ten speech tasks using the BSS label. Our proposed framework is compared with three baselines: eGeMAPs + SVM (traditional machine learning), Qwen-few-shot (general-purpose LLMs), and Wav2Vec 2.0 + BERT (compact self-supervised model). The results are reported in the format of mean and 95% CIs of five random seeds, where error bars represent 95% CIs. For eGeMAPs + SVM, a single run is shown due to deterministic SVM training with no random seed varibility. Statistical significance is determined by the two-sided Wilcoxon signed-rank test with false discovery rate (FDR) correction, where ∗*p* < 0.05, ∗∗*p* < 0.01, and ∗∗∗*p* < 0.001 indicate significance, and “NS” denotes nonsignificant differences. For comparisons with eGeMAPs + SVM, *p* values are calculated using predictions from the median-accuracy seed of each method against eGeMAPs + SVM predictions.

**Figure 3:**
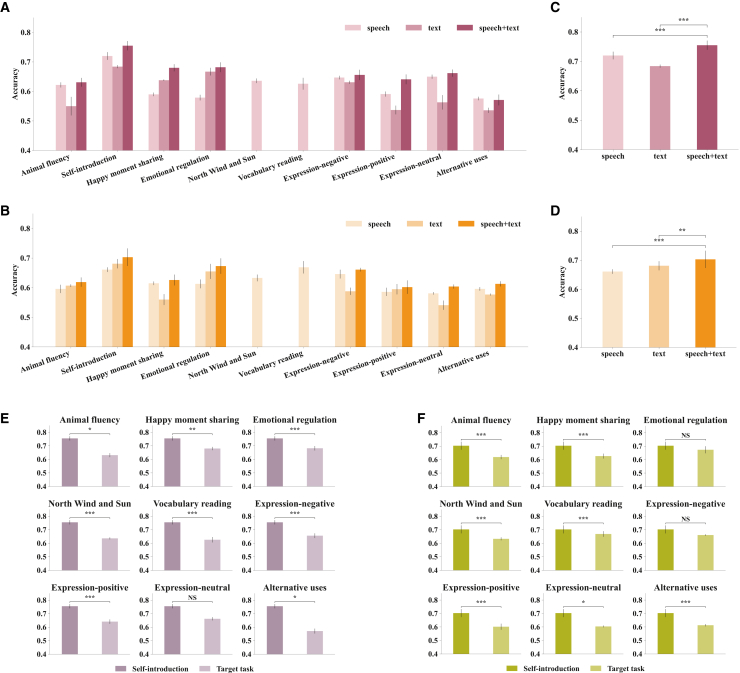
Accuracy of our proposed framework compared with unimodal variants across ten speech tasks using MINI-KID and BSS labels (A) Accuracy of our proposed multimodal (speech + text) model vs. unimodal (speech-only, text-only) models across ten speech tasks using MINI-KID labels. (B) Accuracy of our proposed multimodal (speech + text) model vs. unimodal (speech-only, text-only) models across ten speech tasks using BSS labels. (C) Accuracy comparison of our proposed multimodal (speech + text) model and unimodal (speech-only, text-only) models on the *self-introduction* task with MINI-KID labels. (D) Accuracy comparison of our proposed multimodal (speech + text) model and unimodal (speech-only, text-only) models on the *self-introduction* task with BSS labels. (E) Accuracy of our proposed multimodal (speech + text) model on the *self-introduction* task vs. the other nine tasks with MINI-KID labels. (F) Accuracy of our proposed multimodal (speech + text) model on the *self-introduction* task vs. the other nine tasks with BSS labels. The results are reported in the format of mean and 95% CIs of five random seeds, where error bars represent 95% CIs. Statistical significance is determined by the two-sided Wilcoxon signed-rank test with FDR correction, where ∗*p* < 0.05, ∗∗*p* < 0.01, and ∗∗∗*p* < 0.001 indicate significance, and “NS” denotes nonsignificant differences.

The complete research design process and suicide risk detection framework are shown in Figure 1. Initially, multiple detection methods were evaluated using the MINI-KID label and the *self-introduction* task. Table 2 summarizes the performance of various LLMs, fine-tuning approaches, and fusion strategies. The highest-performing single system achieved an average accuracy of 0.755, with the Whisper and Qwen1.5 models combined through parameter-efficient fine-tuning (PEFT) and concatenate fusion. To further contextualize performance, this system was compared against three baseline methods within the *self-introduction* task. As shown in Table 3, the eGeMAPs + support vector machine (SVM) method yielded an accuracy of 0.537, the Qwen-few-shot method achieved 0.585, and the Wav2Vec 2.0 + bidirectional encoder representations from transformers (BERT) method reached 0.616. In contrast, our “Whisper + Qwen” framework provided a significant performance advantage over all baselines (*p* < 0.001; Figure 2A). We further compared performance between modalities, examining “speech + text” fusion versus unimodal “speech” or “text” approaches across the ten tasks (Figure 3A). The “speech + text” fusion achieved superior performance compared to unimodal approaches in most tasks. Notably, the *self-introduction* task yielded the highest mean accuracy for all three modalities, and within this task, the “speech + text” fusion significantly outperformed unimodal framework (*p* < 0.001; Figure 3C). To leverage complementary system strengths, we constructed an ensemble of the top three single systems using a voting strategy. This ensemble achieved the highest overall accuracy (0.808) and macro-F1 score (0.807) for suicide risk detection on the *self-introduction* task (Table 2). Furthermore, subgroup analyses were conducted, and comparable model performance was observed across gender and age groups for the *self-introduction* task (Table S2).

**Table 2:** Results of different tuning and fusion strategies for LLMs on the self-introduction speech task using the MINI-KID label

System ID	Speech	Text		Fusion	Accuracy-avg	Accuracy-max	Macro-F1-avg	Macro-F1-max
	Model	Model	Tuning					
1	whisper	–	–		0.720 (0.707–0.733)	0.741	0.718 (0.704–0.732)	0.740
2	–	Qwen1.5	APFT		0.636 (0.624–0.648)	0.650	0.635 (0.621–0.649)	0.649
3	–	Qwen1.5	PEFT		0.684 (0.679–0.689)	0.692	0.682 (0.675–0.689)	0.691
4	–	Baichuan2	APFT		0.675 (0.661–0.689)	0.683	0.669 (0.652–0.686)	0.680
5	–	Baichuan2	PEFT		0.658 (0.645–0.671)	0.683	0.653 (0.639–0.667)	0.680
6	whisper	Qwen1.5	APFT	CC	0.648 (0.630–0.656)	0.675	0.647 (0.621–0.673)	0.674
**7**	**whisper**	**Qwen1.5**	**PEFT**	**CC**	**0.755 (0.740–0.770)**	**0.775**	**0.753 (0.737–0.769)**	**0.771**
**8**	**whisper**	**Baichuan2**	**APFT**	**CC**	**0.725 (0.709–0.741)**	**0.750**	**0.722 (0.708–0.736)**	**0.744**
9	whisper	Baichuan2	PEFT	CC	0.718 (0.702–0.734)	0.741	0.716 (0.699–0.733)	0.740
10	whisper	Qwen1.5	APFT	IC	0.703 (0.689–0.717)	0.725	0.700 (0.685–0.715)	0.720
**11**	**whisper**	**Qwen1.5**	**PEFT**	**IC**	**0.717 (0.689–0.745)**	**0.775**	**0.712 (0.683–0.741)**	**0.773**
12	whisper	Baichuan2	APFT	IC	0.636 (0.576–0.696)	0.733	0.578 (0.556–0.600)	0.732
13	whisper	Baichuan2	PEFT	IC	0.681 (0.655–0.707)	0.708	0.676 (0.644–0.708)	0.706
**Voting Results**					–	**0.808**	–	**0.807**

APFT, adaptive pre-training and fine-tuning; PEFT, parameter-efficient fine-tuning; CC, concatenation fusion; IC, in-context fusion. The ensemble results of our best systems, System IDs 7, 8, and 11 are listed in the last line and are marked in bold. The results are reported in the format of mean and 95% CIs across five different random seeds.

**Table 3:** Accuracies of different methods across ten speech tasks using the MINI-KID label

	eGeMAPs + SVM	Qwen- few-shot	Wav2Vec 2.0 + BERT			Whisper + Qwen (ours)		
			speech	text	speech + text	speech	text	speech + text
Animal fluency	0.527	–	0.489	–	0.504	0.622	0.550	0.631
			(0.476–0.502)		(0.486–0.522)	(0.614–0.630)	(0.519–0.581)	(0.617–0.645)
Self-introduction	0.537	0.585	0.492	0.612	0.616	0.720	0.684	0.755
		(0.562–0.608)	(0.461–0.523)	(0.589–0.635)	(0.602–0.630)	(0.707–0.733)	(0.679–0.689)	(0.740–0.770)
Happy moment sharing	0.550	0.559	0.615	0.601	0.628	0.590	0.638	0.680
		(0.549–0.569)	(0.609–0.621)	(0.599–0.603)	(0.614–0.642)	(0.584–0.596)	(0.636–0.640)	(0.668–0.692)
Emotional regulation	0.534	0.581	0.515	0.625	0.632	0.579	0.667	0.682
		(0.541–0.621)	(0.501–0.529)	(0.613–0.637)	(0.612–0.652)	(0.570–0.588)	(0.654–0.680)	(0.666–0.698)
North Wind and Sun	0.533	N/A	0.528	N/A	N/A	0.636	N/A	N/A
			(0.509–0.547)			(0.629–0.643)		
Vocabulary reading	0.533	N/A	–	N/A	N/A	0.626	N/A	N/A
						(0.606–0.646)		
Expression-negative	0.516	0.489	0.615	0.630	0.628	0.647	0.631	0.656
		(0.475–0.503)	(0.605–0.625)	(0.623–0.637)	(0.618–0.638)	(0.641–0.653)	(0.626–0.636)	(0.639–0.673)
Expression-positive	0.482	0.499	–	–	–	0.591	0.537	0.641
		(0.478–0.520)				(0.583–0.599)	(0.523–0.551)	(0.625–0.657)
Expression-neutral	0.542	0.526	0.579	0.589	0.581	0.650	0.563	0.662
		(0.493–0.559)	(0.551–0.607)	(0.582–0.596)	(0.570–0.592)	(0.643–0.657)	(0.539–0.587)	(0.650–0.674)
Alternative uses	0.535	0.549	0.490	0.584	0.575	0.576	0.536	0.571
		(0.535–0.563)	(0.450–0.530)	(0.577–0.591)	(0.566–0.584)	(0.570–0.582)	(0.528–0.544)	(0.553–0.589)

For our “Whisper + Qwen” method, we used the best-performing single-system configuration identified in Table 2, specifically system ID 7, which combined Whisper with Qwen1.5 using PEFT tuning and CC fusion. Text modality results for the two reading tasks are marked as N/A. “–” indicates that the model classified all test samples into a certain category in this setting. The results are reported in the format of mean and 95% CIs across five different random seeds.

Validation was then extended to nine additional speech tasks, again comparing our “Whisper + Qwen” framework against the three baselines (Table 3). Across nearly all evaluated tasks, our method consistently outperformed baseline approaches, achieving the highest accuracy in the majority of cases, with statistically significant improvements (*p* < 0.001 in most comparisons; Figure 2A). Notably, across all ten tasks and all evaluated methods, our framework on the *self-introduction* task performed best, achieving an average accuracy of 0.755, whereas the highest average accuracy attained by the three baseline methods across all tasks remained below 0.65. Moreover, when comparing performance across tasks using our framework, the *self-introduction* task demonstrated significantly higher accuracy than nearly all nine tasks (*p* < 0.001 in most comparisons; Figure 3E).

#### Model results derived from BSS label

In addition to the MINI-KID interview for assessing suicide risk, the self-report BSS was employed as an alternative label to further evaluate the adaptability of the proposed method. Using the same setup established with the MINI-KID label, the methods were trained on the *self-introduction* task with the BSS label under three configurations: “Whisper + Qwen1.5 concatenation fusion,” “Whisper + Baichuan2 concatenation fusion,” and “Whisper + Qwen1.5 in-context fusion.” The results are shown in Table S3. Consistent with the results based on the MINI-KID label, the Whisper and Qwen1.5 model with PEFT and concatenate fusion yielded the best single-system performance, achieving an average accuracy of 0.703.

Similarly, experiments were conducted across ten speech tasks to compare the performance of our “Whisper + Qwen” framework with three baseline methods (Table S4). Our framework consistently outperformed the baselines in nearly all tasks, with the *self-introduction* task yielding the strongest results (average accuracy = 0.703; Figure 2B). This pattern was consistent with findings obtained using the MINI-KID label. On the *self-introduction* task specifically, our framework achieved significant gains in average accuracy compared with the three baseline methods, with relative improvements of 17.3%, 9.5%, and 5.7%. Furthermore, when comparing performance across tasks, the *self-introduction* task demonstrated significantly higher accuracy than nearly all nine tasks (*p* < 0.001 in most comparisons; Figure 3F).

**Figure 4:**
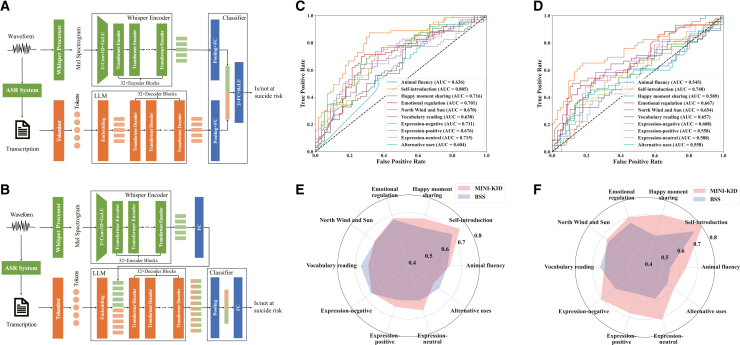
Proposed fusion framework and detection performance across ten speech tasks using MINI-KID and BSS labels (A) Architecture of the proposed framework with concatenation-based fusion. (B) Architecture of the proposed framework with in-context fusion. (C and D) Receiver operating characteristic (ROC) curves and corresponding AUC values for ten speech tasks with MINI-KID and BSS labels. (E and F) Radar plots showing task-wise accuracy and AUC for MINI-KID and BSS labels.

We also examined the performance of different modalities, comparing the “speech + text” fusion with unimodal “speech” or “text” approaches across ten tasks (Figure 3B). Consistent with previous findings based on the MINI-KID label, the fusion modality achieved the highest accuracy on the *self-introduction* task and clearly outperformed unimodal frameworks within this task (*p* < 0.01; Figure 3D). Ensemble voting results for the top three single systems, reported in Table S3, further improved performance, achieving an accuracy of 0.759 and a macro-F1 score of 0.733. Moreover, Figure 4 provides a comprehensive comparison of our proposed framework across ten tasks using both MINI-KID and BSS labels. As shown, the *self-introduction* task consistently produced the best detection performance under both labeling schemes, with particularly stronger results under the MINI-KID label.

### Results from mobile application-based speech data in real-world settings

Building on our finding that our proposed method achieved the optimal detection performance on the *self-introduction* task under the MINI-KID label from voice recorder-based data, we further analyzed this task within a newly developed mobile application to enable external validation in real-world settings. The model previously trained on voice recorder-based data was adapted to mobile application-based recordings using the PEFT strategy. Specifically, the three best-performing single-model configurations (System IDs: 7, 8, and 11 in Table 2) were fine-tuned separately and then combined using a soft-voting ensemble. The corresponding results are reported in Table S5. Overall, the ensemble approach yielded effective performance for suicide risk detection using mobile application-based data, achieving an accuracy of 0.779 and a macro-F1 score of 0.719.

## Discussion

This study indicates that speech analysis integrated with LLMs may offer a feasible and effective method for detecting suicide risk in adolescents. Our framework combines Whisper-Large-v3 speech embeddings with Qwen1.5-7B text embeddings and demonstrates strong performance on the open-ended *self-introduction* task. The voting ensemble achieved an accuracy of 0.808 and a macro-F1 score of 0.807 using the MINI-KID label, and 0.759 and 0.733, respectively, using the BSS label. Across the ten speech tasks, our framework achieved the highest accuracy and AUC on the *self-introduction* task, with statistically significant differences observed in nearly all pairwise comparisons. In addition, our framework significantly outperformed baseline approaches, including eGeMAPs + SVM, Qwen-few-shot, and Wav2Vec 2.0 + BERT. Furthermore, the model adapted effectively from voice recorder-based to mobile application-based recordings, with only a modest reduction in performance (accuracy 0.779; macro-F1 0.719). These findings support the practical potential of LLM-enabled speech analysis for scalable, real-world assessment of suicide risk in adolescents.

The unstructured *self-introduction* task outperforms all other evaluated speech tasks in predicting suicide risk, underscoring the informative value of spontaneous speech in suicide risk assessment. Unlike more constrained tasks, the *self-introduction* prompts adolescents to reflect on themselves from multiple perspectives, encouraging rich emotional expression and self-disclosure.^25^ This is particularly important, given that low self-disclosure has been strongly associated with increased suicidal ideation and behavior,^26^ whereas negative self-referential thinking, which commonly arises from such reflection, is closely linked to suicide risk.^27^ The freedom afforded by the unstructured format allows for the emergence of nuanced acoustic and linguistic features, providing deeper insight into individuals’ internal emotional states. This aligns with prior research emphasizing the diagnostic potential of unstructured speech in capturing authentic emotional expression and self-disclosure, which may be crucial for identifying suicidal intent.^5^ Building on the self-referential reflection and disclosure elicited by the *self-introduction* task, an important next step is to clarify whether the most predictive acoustic and semantic features index neurobiological processes implicated in suicide risk. Future work integrating speech sampling with neuroimaging and genetic risk indices can systematically evaluate these mechanism-linked interpretations and further strengthen the construct specificity of speech-based markers.

In contrast, other tasks, including Passage Reading, Vocabulary Reading, Animal Fluency Test, Emotional Face Description, Alternative Uses, happy moment sharing, and emotional regulation, demonstrated weaker predictive performance. This can largely be attributed to their constrained formats and limited capacity to evoke spontaneous or emotionally rich responses.^28^ For example, reading tasks provide only acoustic input, omitting semantic and higher-order cognitive signals.^29^ Although tasks such as emotional regulation and happy moment sharing incorporate both verbal and non-verbal features, their structured nature often elicits superficial or formulaic responses, restricting emotional depth and self-disclosure.^30^^,^^31^ Thus, despite their multimodal design, these tasks fail to provide the same depth of emotional expression and self-disclosure that the *self-introduction* task facilitates.

Adolescence is a developmental period marked by substantial maturation in speech production and language use. Age-related differences in vocabulary breadth, narrative organization, and typical response length across 10–18 years may influence both transcript-derived representations and paralinguistic features.^32^ The gender composition of the sample may also affect generalizability, given potential differences in communication style and disclosure patterns.^33^ Although we conducted age-band and gender-stratified analyses and observed broadly comparable performance across strata, these developmental and demographic factors remain important considerations for interpretation and deployment. The study context also informs cross-language generalizability. Text-based components that depend on transcripts and LLM-derived semantics are language dependent and may reflect culturally shaped norms of expression, whereas several acoustic dimensions, including prosody and voice quality, are comparatively less language bound, but may still be sensitive to phonology and recording context.^34^ Cross-language transportability should therefore be tested empirically using multilingual cohorts with local calibration. Moreover, speech collected within a fixed assessment window and a specific elicitation context is best interpreted as indexing a current risk-related state. This framing motivates longitudinal monitoring to distinguish transient distress from sustained high-risk trajectories and to increase the clinical actionability of repeated assessments over time. In this context, integrating speech with long-timescale wearable sensor signals can further strengthen a multimodal digital phenotyping framework for suicide risk.^35^ By jointly capturing expressive behavior and autonomic dynamics, such integration may improve sensitivity to proximal risk fluctuations and support more actionable longitudinal monitoring with timely follow-up.

Furthermore, in comparison to prior studies using traditional machine learning methods, our LLM-based framework shows significantly enhanced performance in detecting suicide risk from spontaneous speech. Previous efforts, such as those by Belouali et al.^8^ and Tlachac et al.,^10^ relied on handcrafted acoustic features combined with conventional classifiers, achieving moderate performance (AUC 0.78; balanced accuracy 0.73). However, applying similar models (e.g., eGeMAPS + SVM) to our data yielded accuracies of only ∼50%, indicating their limited capacity to detect subtle emotional and semantic cues in spontaneous speech.^5^^,^^6^ In contrast, our LLM-based framework, pre-trained on large corpora and fine-tuned for downstream tasks, effectively integrate acoustic and linguistic information, yielding more accurate and context-aware predictions.^17^ These findings not only validate the effectiveness of the unstructured *self-introduction* task but also emphasize the advantages of LLMs for scalable, automated suicide risk detection.

Our framework also demonstrates consistent performance when trained and evaluated on two different suicide assessment tools: the MINI-KID interview (clinician-administered) and the BSS self-report. By training models separately, the framework demonstrates flexibility in handling heterogeneity across data sources.^36^ This illustrates the potential for generalizability across different clinical assessment types, provided that each dataset is appropriately handled and evaluated. Furthermore, our findings highlight the generalizability of the proposed method when transitioning from constrained laboratory conditions to real-world applications. By leveraging fine-tuning and a soft-voting ensemble strategy, our framework effectively mitigated the domain discrepancy between standardized interview settings and real-world environments. This domain adaptation resulted in effective detection performance across settings, underscoring the framework’s translational value and enhancing its practical utility in diverse contexts.

In practice, the model output should be interpreted as a decision-support signal that prioritizes timely follow-up, rather than a stand-alone diagnostic determination. Within a school screening workflow, a positive prediction is best treated as an indication for a brief secondary assessment by trained staff, with escalation to clinical evaluation when warranted. False negatives remain possible and underscore that automated outputs should not override direct disclosure, observed behavioral concern, or established safety procedures. Implementation in screening settings requires prespecifying an operating point that aligns with clinical and school resources.^37^ In many preventive settings, sensitivity-prioritized operating points may be appropriate when paired with feasible follow-up capacity and clear safeguards. Importantly, our model was trained and evaluated in cohorts enriched through screening to support initial validation. Performance may therefore differ when the same model is applied to an unselected, broader school population with a lower base rate of suicide risk.^38^ Prospective evaluation in representative school cohorts, together with calibration to local prevalence and operational capacity, will be essential before routine deployment.

### Limitations of the study

Despite the effective detection performance of our framework, several limitations warrant consideration. First, the gender distribution in our samples is skewed toward female participants. Although gender-stratified analyses yielded comparable model performance, this imbalance may still limit the generalizability of the findings across gender groups. Second, the speech dataset collected in our interview setting reflects a more balanced class distribution, and models trained on such samples may exhibit reduced performance in real populations with lower prevalence. This tendency was also observable in our mobile application-based data collected in real-world settings, where the lower prevalence was accompanied by a modest reduction in performance. Moreover, the generalizability of our findings to other languages and cultural contexts remains uncertain and warrants further investigation. To ensure the equitable applicability of our proposed framework, future studies should prioritize the development of multilingual and cross-cultural datasets.

## Resource availability

### Lead contact

For further information and requests for resources, please contact the lead contact, Runsen Chen (runsenchen@tsinghua.edu.cn).

### Materials availability

This study did not generate new unique reagents.

### Data and code availability

• Data: All data reported in this paper will be shared by the lead contact upon reasonable request.
• Code: The code used for data processing and model development is publicly available at https://github.com/cuiziyun/suicide_speech.
• Any additional information required to reanalyze the data reported in this paper is available from the lead contact upon request.

## Acknowledgments

This study was supported by the New Generation Artificial Intelligence - National Science and Technology Major Project of China (no. 2025ZD0121803), National Natural Science Foundation of China (no. 62476151), Beijing Natural Science Foundation (no. L243036), Beijing, China, and Research Fund of Vanke School of Public Health, Tsinghua University, Beijing, China.

## Author contributions

C.L., Z.C., C.Z., and R.C. designed the research; C.L., Y.D., D.Q., and Z.Z. performed the research; B.Z. and J.W. contributed new analytic tools; C.L., Z.C., W.W., and C.Z. analyzed the data; J.S.J. provided comments on the paper; and C.L., Z.C., Z.W., D.Q., C.Z., and R.C. wrote and revised the paper.

## Declaration of interests

The authors declare no competing interests.

## STAR★Methods

### Key resources table

**Table:** 

REAGENT or RESOURCE	SOURCE	IDENTIFIER
**Software and algorithms**		

Python version 3.10	Python	https://www.python.org/
Pytorch version 2.1.0	Pytorch	https://pytorch.org/
Transformers version 4.38.1	Transformers	https://huggingface.co/docs/transformers
PEFT version 0.11.1	Mangrulkar et al.	https://github.com/huggingface/peft
Sox	Bagwell et al.	http://sox.sourceforge.net
Whisper-Large-v3	Radford et al.	https://huggingface.co/openai/whisper-large-v3
Baichuan2-7B-Base	Baichuan Intelligence Inc.	https://huggingface.co/baichuan-inc/Baichuan2-7B-Base
Qwen1.5-7B	Bai et al.	https://huggingface.co/Qwen/Qwen1.5-7B
Source code	GitHub	https://github.com/cuiziyun/suicide_speech

**Other**		

NVIDIA A800 GPU	NVIDIA	https://www.nvidia.com/

### Experimental model and study participant details

Speech data were collected from adolescents aged 10 to 18 years as part of a school-based mental health screening program in Yunfu, Guangdong Province, China. The collection process was conducted in two phases: the first phase involved gathering voice data under controlled experimental conditions and included 1,223 participants from 47 schools, whereas the second phase focused on the development of a mobile application and the collection of real-time speech data, encompassing 460 participants from 14 schools. Detailed information on participant age and gender is presented in Table 1. The analyses in this study explicitly evaluate performance across age and gender, as reported in the Results and Table S2.

For both phases of speech collection, participants first underwent a mental health screening in the school computer room, during which they self-reported any lifetime history of self-harm. Adolescents who endorsed such a history were invited to participate in the study. Those who were further identified as having current suicide risk on the MINI-KID interview or current suicidal ideation on the BSS were subsequently invited to participate in the speech assessment. Additionally, a control group was included for comparison. Participants in this group were confirmed to have no current suicide risk or suicidal ideation and underwent the same assessment procedures and speech recording protocol. Informed consent was obtained from all participants and their guardians before any data collection, and the study protocol was approved by the Tsinghua University Institutional Review Board.

In the first phase, speech recordings were conducted in a quiet, soundproof room to minimize background noise. Only the interviewer and participant were present, thereby ensuring privacy and standardized administration. Standardized procedures were implemented to maintain consistency across recording conditions. This phase included ten speech tasks, encompassing both unstructured and structured formats. All interviewers used identical voice recorders to maintain consistency in audio format and recording quality across participants.

While the first phase ensured high-quality recordings in a controlled setting, its ecological validity and real-world applicability were limited. To this end, we developed a mobile application for real-time speech data collection, enabling participants to complete speech recordings using the mobile phones. While this phase included a diverse range of structured and unstructured speech tasks, the analysis focused exclusively on the unstructured *self-introduction* task. This decision was guided by the predictive validity observed across tasks in the first phase. This phase not only served to validate the practicality of mobile application-based speech collection but also provided critical insights into its usability and acceptability within the real-world environment.

### Method details

#### Speech task measures

The speech data comprised responses to ten distinct tasks, strategically designed to capture a comprehensive spectrum of linguistic, cognitive, and emotional features relevant to suicide risk assessment. In the first phase, all ten tasks were administered using a voice recorder to enable a comprehensive evaluation of speech-based indicators. In contrast, the second phase, leveraging a mobile application for real-time speech data collection, focused exclusively on the unstructured *self-introduction* task. This targeted approach was informed by insights from the first phase, prioritizing both predictive validity and consistency in real-time data acquisition. A detailed description of each speech task is provided, and the full data collection process is illustrated in Figure S1.

1) Task 1: This task utilized the *Animal Fluency Test*, traditionally a measure of cognitive executive function but also a key component in language processing.^39^ Participants were asked to list as many animals as possible, revealing cognitive and linguistic abilities.
2-4) Tasks 2 to 4 Unstructured Question Sessions: These tasks involved three open-ended questions including *self-introduction*, *happy moment sharing* and *emotional regulation*, for example, *How would you describe yourself? Please evaluate yourself from different perspectives. (self-introduction); Share with us your happiest or proudest memory. Could you describe the scene at that time? (happy moment sharing); Have you ever experienced moments of extreme emotional distress? How do you manage such feelings? (emotional regulation)*.
5) Task 5 Passage Reading: Participants read the poetry: *The North Wind and Sun*, a passage from Aesop’s Fables commonly used in linguistic studies for examining structures such as morphemes.^40^
6) Task 6 Vocabulary Reading: This task involved reading a list of positive (e.g., passion, outstanding), negative (e.g., shame, inferiority), and neutral (e.g., commodity, center) words. Positive and negative words were selected from the affective ontology corpus,^41^ and neutral ones were picked out from the Chinese affective words’ extremum table.^42^
7-9) Tasks 7–9 Emotional Face Description: Participants were shown images of faces displaying positive, neutral, and negative emotions and asked to describe each one. Three adult Asian face emotion expression images were selected from the racially diverse affective expression (RADIATE) face stimulus set.^43^
10) Task 10 *Alternative Uses* task: In this task, participants described possible uses for an *Empty Box.* The *Alternative Uses* task is widely used in studies of divergent thinking and creativity.^44^ Similar to the *Animal Fluency Test*, responses in the *Alternative Uses* task revealed not only acoustic and linguistic features but also cognitive traits relevant to suicide risk assessment.

#### Suicide risk assessment: Procedures and validation

The MINI-KID is a brief, structured diagnostic interview designed for adolescent, validated for use by clinicians and researchers to aid in diagnosing common psychiatric disorders based on DSM-IV and ICD-10 criteria.^45^ For this study, we utilized the suicide module of the MINI-KID, which includes questions formatted in a binary yes/no format. According to the standardized scoring and administration procedures of this module, participants were classified as either currently at risk of suicide or not currently at risk of suicide. The MINI-KID is considered a gold standard interview for assessing adolescent psychiatric disorders and has been utilized for assessing current suicide risk using a one-month evaluation window, incorporating three dimensions: suicidal ideation, specific planning, and actual attempts.^46^ The validated Chinese version of the MINI-KID has demonstrated acceptable reliability and validity in Chinese adolescents,^47^ and it is routinely used in both clinical and research settings.^48^^,^^49^^,^^50^

In our study, all interviewers completed a standardized training program. Following training, the interviewers demonstrated high reliability, achieving an overall diagnostic agreement of 97.7% with the gold standard ratings. Inter-rater reliability for MINI-KID diagnoses was similarly strong, with a Fleiss κ of 0.894 (95% confidence interval 0.88 to 0.91).

The BSS is a self-report measure consisting of 21 items rated on a 3-point Likert scale.^51^ It assesses the intensity, pervasiveness, and characteristics of suicidal ideation. In this study, we used items 1–19, as these specifically address suicidal ideation experienced in the past week. Based on the scoring criteria, participants were then classified as having current suicidal ideation or not. The BSS is widely used in suicide research, and acceptable reliability and validity have been demonstrated.^52^ The Chinese version of the BSS has shown good psychometric properties in adolescent samples^53^ and has been applied in large school-based studies in China.^54^ In our study, internal consistency of the BSS was high (Cronbach’s α = 0.924), indicating good reliability of the measure.

These two tools were selected to provide complementary assessments: the MINI-KID offers a clinician-administered diagnostic evaluation of suicide risk over a broader time frame (past month), while the BSS provides more fine-grained, self-reported insights into suicidal ideation within a shorter window (past week). Therefore, we independently trained and evaluated our approach on datasets labeled by each tool. This strategy ensured that approach performance could be interpreted within the appropriate clinical context for each assessment type.

#### Mobile application platform for speech data collection

We developed a mobile application to facilitate real-time speech data collection. The application was designed with a user-friendly interface, enabling participants to complete the speech tasks independently on the mobile phones. Participants were only asked to install and use the application after providing informed consent, which was obtained before any data collection began. The interface featured a clear, intuitive layout with easy navigation to minimize participant burden and maximize usability.

To ensure data security and confidentiality, the application incorporated robust encryption protocols for both data storage and transmission. All speech data collected through the application was securely uploaded to a centralized cloud server, accessible only by authorized research personnel. The data upload process was designed to operate efficiently, even with varying internet connectivity, ensuring reliable transmission without compromising user experience. Ethical considerations were prioritized throughout the development, ensuring participants understood the purpose of data collection and the measures in place to protect their privacy. A sample of the application interface can be found in Figure S2.

#### Crisis support procedures

Participants were informed of their right to withdraw from the study at any time and to decline to answer any questions they found uncomfortable. Participants identified as being at risk on the basis of the MINI-KID interview received appropriate crisis support in accordance with local and school policies. Such support could include referral to school mental health teachers or school emergency teams, safety planning, involvement of teachers and parents or guardians, and referral for further psychiatric evaluation or mental health services when warranted. All participants were also provided with psychoeducational materials and information on crisis and mental health services.

#### Preprocessing of speech data

The voice recorder-based speech data in the first phase was divided into training, validation, and test sets (8:1:1), ensuring participant-level partitioning to prevent overlap-related performance inflation. To enhance voice content density, recordings were segmented into utterances using the voice activity detection (VAD) module,^55^ removing pauses. Recordings under five seconds post-VAD were excluded due to low informational content. Table S1 presents the number and total duration (in hours) of recordings for each speech task on voice recorder-based speech data, including both original and processed data. The total original duration was 146.3 h. After preprocessing, there were 83.8 h for the MINI-KID label and 82.0 h for the BSS label.

In contrast, mobile application-based speech data faced greater variability and noise, complicating task completion. To address this, voice enhancement and VAD-based silence removal were applied, ensuring consistency with the first phase preprocessing pipeline. This approach optimized data quality while accounting for environmental variability. The total duration of speech recordings collected for the *self-introduction* task was 317 min (5.3 h). After preprocessing, the total duration was reduced to 184 min (3.1 h).

#### Development of the detection model

The model’s overall architecture is illustrated in Figure 1 and consists of two main branches: a speech branch and a text branch. The speech branch extracts relevant features from audio using the Whisper-Large-v3 model,^56^ a Transformer-based encoder-decoder model, with 640 million (M) parameters in the encoder, pre-trained on 1M hours of weakly labeled audio and 4M hours of pseudo-labelled audio for ASR tasks, allowing its encoder to generate robust representations of speech content. The text branch processes transcribed text using LLMs, Baichuan2-7B^57^ and Qwen1.5-7B,^58^ which are both Transformer-based, decoder-only models with 7 billion (B) parameters each, pre-trained on over 2 trillion tokens of Chinese and English data. These features are then combined for classification, where two fusion strategies were investigated. One is concatenate fusion, a feature-level fusion where each backbone model independently extracts modality-specific features, which are then pooled, concatenated, and fed into a classifier to assess suicide risk.^59^^,^^60^ Another is in-context fusion, inspired by modality aliment mechanisms in multimodal LLMs,^61^ where speech embeddings are projected from the speech hidden space into the LLMs hidden space via a fully connected layer, serving as additional context tokens, allowing the LLMs to perform multimodal fusion through its contextual reasoning capability. The fused features are finally classified to assess suicide risk. The detailed structure of our proposed framework is illustrated in Figure 4, showcasing two fusion strategies: concatenate fusion (Figure 4A) and in-context fusion (Figure 4B).

In addition, we apply transfer learning to adapt these models for suicide risk detection, leveraging their extensive general capabilities from large-scale pre-training and fine-tuning them on our specialized dataset to capture features relevant to the specific task. Two approaches are used: Adaptive Pre-training and Fine-Tuning (APFT), which updates all model parameters, and Parameter-Efficient Fine-Tuning (PEFT), which uses the Low-Rank Adaptation (LoRA)^62^ method to reduce computational load. Beyond evaluating each single system separately, we also constructed an ensemble to leverage the complementary strengths of diverse LLMs-based models.^63^ After training all candidate systems with different LLMs backbones and fusion strategies, we selected several best-performing single systems, and conducted a voting scheme to obtain the final ensemble detection. Without introducing additional training, this approach aggregates the predictions of diverse models whose errors are only partially overlapping, thereby improving robustness. Furthermore, our proposed method is compared to three baseline methods, eGeMAPs + SVM^64^ (a traditional machine learning approach), Qwen-few-shot (using a general LLMs directly), and Wav2Vec 2.0 + BERT^65^^,^^66^ (smaller self-supervised learning model), to demonstrate the effective performance of our proposed approach.

#### Adaptation of the model for mobile application-based speech data

After training on voice recorder-based speech data, the model was further adapted to mobile application-based recordings. Unlike the initial training phase, which consisted of two separate stages including fine-tuning the backbone model and training the classifier head, the adaptation stage involves joint training of both components. Given that this stage was intended solely to adapt the previously trained model from voice recorder-based to mobile application-based speech data, rather than training from scratch, PEFT finetuning was employed, adding trainable Weight-Decomposed Low-Rank Adaptation (DoRA)^67^ module to the backbone model. DoRA is a variant of LoRA that decomposes weight updates into magnitude and direction, and has been shown to exhibit update characteristics intermediate between full fine-tuning and standard LoRA. This property makes DoRA well suited to joint training settings in which multiple components need to be optimized simultaneously.

#### Implementation details

The framework was implemented using the *PyTorch*. During fine-tuning of the speech base model, speed perturbation was applied for data augmentation, introducing random variations to the original data to improve model generalization. We used the *Sox* toolkit to adjust speech speed to 0.9× and 1.1× the original, while maintaining pitch, which is crucial for accurate suicide risk detection. For Whisper fine-tuning, audio recordings were segmented into 30 s clips with a window shift of 10 s. Pre-trained models were fine-tuned for 10 epochs on the training set, as well as the classifier. Learning rates for APFT were selected based on the validation set performance, with choices of 1 × 10^−6^, 3 × 10^−6^, and 1 × 10^−5^. For PEFT, learning rates of 1 × 10^−5^, 3 × 10^−5^, and 1 × 10^−4^ were considered. The classifier learning rates were set at 1 × 10^−5^, 1 × 10^−4^, and 1 × 10^−3^. A linear scheduler with a warmup period covering 20% of the total steps was used for learning rate adjustment. Implementation details for the three baseline models are also provided below.

##### eGeMAPs + SVM

We extracted the 88-dimensional eGeMAPs acoustic feature set (covering frequency, energy, spectral, and temporal descriptors), and trained an SVM classifier for suicide risk detection. Hyperparameters were tuned per speech task with grid search on the validation set, and the best-performing model was used to evaluate the test set.

##### Qwen-few-shot

We evaluated an LLM few-shot baseline using Qwen1.5-7B-Chat. For each test instance, the model received a prompt containing four training examples followed by the test transcript (prompt format in Figure S3) and generated a suicide-risk assessment; generation temperature was set to 1. To reduce prompt-sampling variance, we repeated experiments five times with different randomly selected in-context examples and reported averaged performance.

##### Wav2Vec 2.0 + BERT

As a self-supervised learning (SSL) baseline for Chinese data, we used Wav2Vec2-XLSR-53 for speech representations and BERT-Base-Chinese for text representations, then performed concatenate fusion for classification following our overall pipeline. To leverage information across Transformer depths, we formed utterance representations using a trainable weighted sum of multiple layer features rather than selecting a single layer. Models were fine-tuned on the training set with APFT, trained separately per speech task, with learning rates selected on validation performance. We ran five random seeds and reported averaged results.

### Quantification and statistical analysis

Model performance was evaluated on the test set, with configurations optimized on the validation set. To ensure reliable results and minimize randomness, five independent experiments were conducted with different random seeds for each configuration, with the results reported as mean and 95% confidence intervals (CIs). The evaluation metrics used include accuracy, macro-F1 score and area under the ROC curve (AUC). Accuracy is the proportion of correctly classified participants, macro-F1 is the average F1 score across categories, giving equal weight to each category, and the AUC summarize the model’s ability to distinguish across decision thresholds. Statistical significance between model performances was assessed using a two-sided Wilcoxon signed-rank test, applied at the sample-level by comparing the prediction categories of paired models for each individual for comparisons involving baseline methods eGeMAPs + SVM and Qwen-few-shot, which yield only categorical predictions without probabilistic outputs, and comparing probabilities of paired models for other comparisons. False Discovery Rate (FDR) correction was applied to the resulting *p*-values to account for multiple comparisons using the Benjamini-Hochberg procedure. Asterisks indicate statistical significance as determined by the two-sided Wilcoxon signed-rank test with FDR correction. Specifically, ∗ corresponds to *p* < 0.05, ∗∗ to *p* < 0.01, and ∗∗∗ to *p* < 0.001. “NS” denotes nonsignificant differences.

Initial experiments investigated a range of model architectures and fusion strategies, leveraging the *self-introduction* speech task annotated with the MINI-KID label, with the objective of identifying the most effective model configuration for suicide risk detection. Building on these preliminary findings, the optimal model was independently applied to each speech task using separate training and evaluation procedures, thereby enabling a systematic assessment of the relative relevance and predictive utility of each task in identifying suicide risk. This comparative analysis further facilitated the identification of the task with the highest predictive accuracy. Additionally, current suicide risk from the MINI-KID interviews and current suicidal ideation from the BSS self-report were used as independent label to evaluate the approach’s adaptability.

## Supplemental information

Document S1. Figures S1–S3 and Tables S1–S5

Document S2. Article plus supplemental information
