# Supplementary material for: Integrating speech biomarkers and large language models for adolescent suicide risk detection with mobile application for real-world evaluation
Source: Cell Rep Med. 2026 Jun 16;7(6):102823. doi: 10.1016/j.xcrm.2026.102823 (PMC13293961; doi:10.1016/j.xcrm.2026.102823)
Supplement: Document S1. Figures S1–S3 and Tables S1–S5 [file mmc1.pdf]

Cell Reports Medicine, Volume *7*

Supplemental information

Integrating speech biomarkers and large language models for adolescent suicide risk detection with mobile application for real-world evaluation

Chang Lei, Ziyun Cui, Yinan Duan, Zhijun Wu, Diyang Qu, Wen Wu, Zeming Zhang, John S. Ji, Bowen Zhou, Ji Wu, Chao Zhang, and Runsen Chen

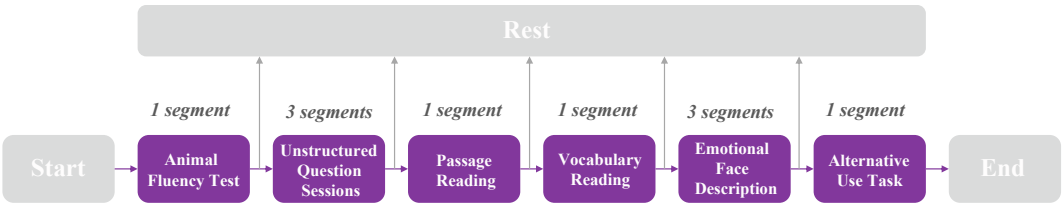

Figure S1. The complete process of collecting speech data. Related to STAR Methods. *The process consists of six units, with a total of ten speech tasks.*

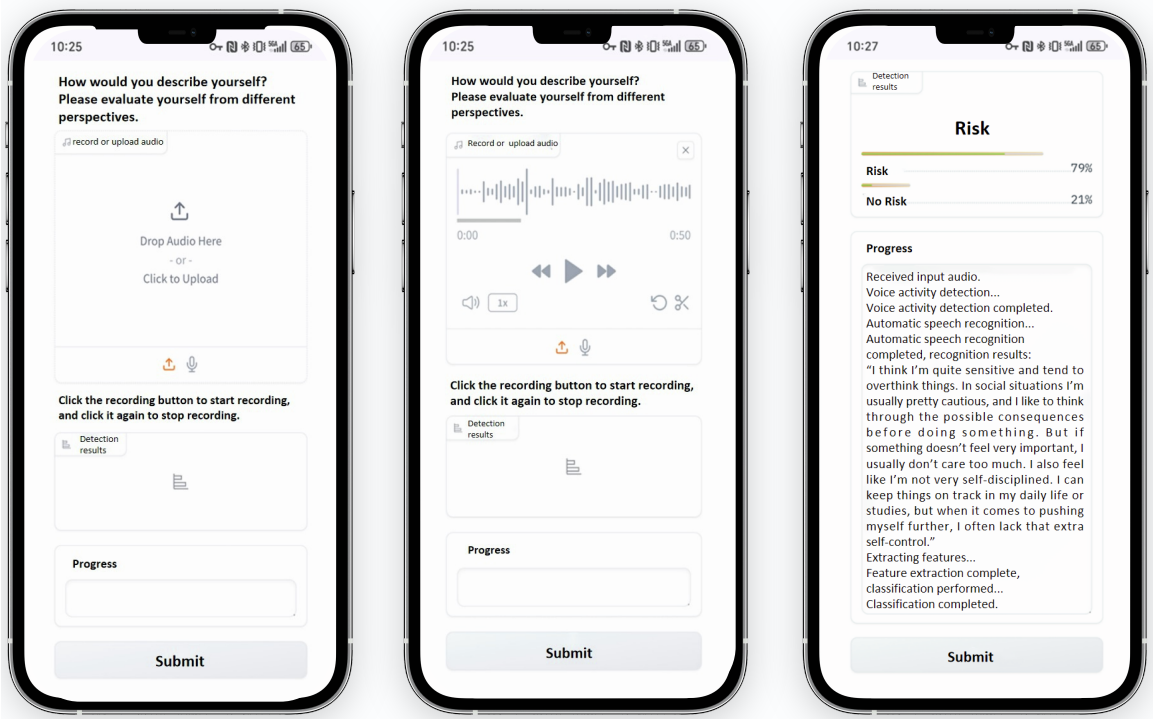

A. User Interface (unfinished) **B. User Interface (finished)** **C. Detection Results**

Figure S2. A sample of the application interface and suicide risk detection process in the mobile application. Related to Figure 1 and STAR Methods.

SYSTEM: You are a professional clinical psychiatrist who is conducting suicide risk assessments for primary and secondary school students.

USER: The question you ask them is "{question description}". Now that you've got a transcript of their speech through Whisper model, you'll make your diagnosis. ONLY answer YES or NO, no other responses. Answer: {Example without suicide risk}

ASSISTANT: NO

USER: Answer: {Example with suicide risk}

ASSISTANT: YES

USER: Answer: {Example without suicide risk}

ASSISTANT: NO

USER: Answer: {Example with suicide risk}

ASSISTANT: YES

USER: Answer: {Sample to test}

ASSISTANT:

Figure S3. Prompt for Qwen-few-shot. Related to STAR Methods. *Originally written in Chinese and translated to English here.*

Table S1. Speech recording number and duration (in hours) for each speech task on voice recorder speech data. Related to STAR Methods.

	MINI-KID label				BSS label			
	Original Recording	Original Duration	Processed Recording	Processed Duration	Original Recording	Original Duration	Processed Recording	Processed Duration
Animal fluency	1166	21.4	1154	11.6	1163	21.3	1151	11.6
Self-introduction	1192	15.8	1179	8.9	1165	15.5	1151	8.6
Happy moment sharing	1191	12.1	1175	7.8	1164	11.8	1148	7.6
Emotional regulation	1194	11.3	1171	7.6	1165	11.0	1142	7.4
North Wind and Sun	1194	16.0	1193	12.8	1167	15.7	1166	12.5
Vocabulary reading	1193	7.3	1193	5.8	1166	7.2	1166	5.6
Expression-positive	1193	7.5	1188	4.2	1166	7.4	1161	4.1
Expression-negative	1194	6.7	1181	3.9	1167	6.6	1154	3.8
Expression-neutral	1194	7.7	1177	4.4	1167	7.5	1150	4.3
Alternative uses	1193	40.5	1157	16.8	1166	40.0	1130	16.5
Total	11904	146.3	11768	83.8	11656	144.0	11519	82.0

Table S2. Subgroup analyses by gender and age for detection performance on the self-introduction speech task on the MINI-KID label. Related to Tables 1 and 2.

	Speech Model	Text Model	Text Tuning	Fusion	Accuracy	Macro-F1
**Male**	Whisper	Qwen1.5	PEFT	CC	0.776 (0.766-0.786)	0.758 (0.748-0.769)
	Whisper	Baichuan2	APFT	CC	0.710 (0.674-0.745)	0.703 (0.666-0.740)
	Whisper	Qwen1.5	PEFT	IC	0.729 (0.688-0.769)	0.701 (0.644-0.757)
	**Voting Results**				**0.833**	**0.816**
**Female**	Whisper	Qwen1.5	PEFT	CC	0.744 (0.725-0.762)	0.717 (0.693-0.741)
	Whisper	Baichuan2	APFT	CC	0.733 (0.720-0.746)	0.697 (0.679-0.715)
	Whisper	Qwen1.5	PEFT	IC	0.710 (0.678-0.743)	0.676 (0.633-0.720)
	**Voting Results**				**0.795**	**0.780**
**Age (10-12)**	Whisper	Qwen1.5	PEFT	CC	0.746 (0.730-0.763)	0.724 (0.702-0.747)
	Whisper	Baichuan2	APFT	CC	0.792 (0.765-0.819)	0.782 (0.753-0.811)
	Whisper	Qwen1.5	PEFT	IC	0.585 (0.527-0.643)	0.568 (0.511-0.625)
	**Voting Results**				**0.808**	**0.793**
**Age (13-18)**	Whisper	Qwen1.5	PEFT	CC	0.757 (0.741-0.773)	0.746 (0.725-0.767)
	Whisper	Baichuan2	APFT	CC	0.706 (0.694-0.719)	0.682 (0.664-0.699)
	Whisper	Qwen1.5	PEFT	IC	0.753 (0.725-0.782)	0.743 (0.711-0.775)
	**Voting Results**				**0.809**	**0.804**

APFT: Adaptive Pre-training and Fine-Tuning; PEFT: Parameter-Efficient Fine-Tuning; CC: concatenation fusion; IC: in-context fusion. The configurations are the same as those used for System IDs 7, 8, and 11 in Table 2. The ensemble results of our best systems are listed in the last line, which are marked in bold. The best single-system configuration was the same as the one reported in Table 2 under System ID: 7, which used Whisper + Qwen1.5, PEFT tuning, and CC fusion. Adolescents aged 10 to 12 were in primary school, whereas those aged 13 to 18 were in secondary school. The results are reported in format of mean and 95% CIs on five different random seeds.

Table S3. Results of different tuning and fusion strategies for LLMs with the self-introduction speech task on the BSS label. Related to Tables 1 and 2.

System ID	Speech	Text	Tuning	Fusion	Accuracy-avg	Accuracy-max	Macro-F1-avg	Macro-F1-max
**7**	Whisper	Qwen1.5	PEFT	CC	0.703 (0.674-0.732)	0.750	0.689 (0.659-0.719)	0.740
**8**	Whisper	Baichuan2	APFT	CC	0.661 (0.630-0.692)	0.694	0.638 (0.614-0.662)	0.662
**11**	Whisper	Qwen1.5	PEFT	IC	0.661 (0.652-0.670)	0.675	0.639 (0.619-0.659)	0.664
**Voting Results**					-	**0.759**	-	**0.733**

APFT: Adaptive Pre-training and Fine-Tuning; PEFT: Parameter-Efficient Fine-Tuning; CC: concatenation fusion; IC: in-context fusion. The configurations are the same as those used for System IDs 7, 8, and 11 in Table 2. The ensemble results of our best systems are listed in the last line, which are marked in bold. The best single-system configuration was the same as the one reported in Table 2 under System ID: 7, which used Whisper + Qwen1.5, PEFT tuning, and CC fusion. The results are reported in format of mean and 95% CIs on five different random seeds.

Table S4. Accuracies of different methods with ten speech tasks on the BSS label. Related to Figures 2, 3, and 4.

	eGeMAPs + SVM	Qwen-few-shot	Wav2Vec 2.0 + BERT			Whisper + Qwen (ours)		
			speech	text	speech + text	speech	text	speech + text
Animal fluency	0.600	–	0.541 (0.520-0.562)	–	0.516 (0.474-0.558)	0.596 (0.582-0.610)	0.607 (0.603-0.611)	0.619 (0.604-0.634)
Self-introduction	0.530	0.608 (0.583-0.633)	0.570 (0.534-0.606)	0.631 (0.575-0.687)	0.646 (0.644-0.648)	0.661 (0.654-0.668)	0.681 (0.666-0.696)	0.703 (0.674-0.732)
Happy moment sharing	0.563	0.497 (0.669-0.525)	0.535 (0.480-0.590)	0.583 (0.574-0.592)	0.594 (0.583-0.607)	0.615 (0.609-0.621)	0.560 (0.542-0.578)	0.626 (0.608-0.644)
Emotional regulation	0.604	0.547 (0.510-0.584)	0.517 (0.460-0.574)	0.650 (0.636-0.664)	0.648 (0.633-0.663)	0.613 (0.599-0.627)	0.655 (0.630-0.680)	0.673 (0.647-0.699)
North Wind and Sun	0.582	N/A	0.525 (0.485-0.565)	N/A	N/A	0.633 (0.622-0.644)	N/A	N/A
Vocabulary reading	0.581	N/A	0.566 (0.543-0.589)	N/A	N/A	0.669 (0.648-0.690)	N/A	N/A
Expression-negative	0.545	0.515 (0.485-0.545)	0.557 (0.491-0.623)	0.682 (0.668-0.696)	0.682 (0.677-0.687)	0.646 (0.632-0.660)	0.588 (0.576-0.600)	0.661 (0.655-0.667)
Expression-positive	0.566	0.476 (0.432-0.520)	–	0.563 (0.549-0.559)	0.565 (0.550-0.580)	0.586 (0.572-0.600)	0.595 (0.578-0.612)	0.602 (0.580-0.624)
Expression-neutral	0.574	0.489 (0.458-0.520)	0.561 (0.554-0.568)	0.616 (0.599-0.633)	0.602 (0.595-0.609)	0.581 (0.577-0.585)	0.541 (0.526-0.556)	0.604 (0.597-0.611)
Alternative uses	0.549	0.535 (0.520-0.550)	0.547 (0.494-0.600)	0.539 (0.520-0.558)	0.554 (0.543-0.565)	0.596 (0.590-0.602)	0.577 (0.574-0.580)	0.613 (0.605-0.621)

For our "Whisper + Qwen" method, we used the best-performing single-system configuration identified in Table S2, specifically System ID: 7, which combined Whisper with Qwen1.5 using PEFT tuning and CC fusion. Text modality results for the two reading tasks are marked as N/A. The "–" refers to that, the model classified all test samples to a certain category in this setting. The results are reported in format of mean and 95% CIs on five different random seeds.

Table S5. Results of model adaptation on mobile application speech data. Related to Table 2 and STAR Methods.

System ID	7	8	11	Ensemble
Accuracy	0.726	0.747	0.663	**0.779**
Macro-F1	0.627	0.696	0.576	**0.719**

Models previously trained on voice recorder speech data (System ID: 7, 8, and 11 in Table 2) were individually adapted and subsequently combined into an ensemble. Accuracy and macro-F1 score on the application-collected test set are listed. In contrast to experiments on voice recorder speech data, the adaptation experiments were conducted with a single random seed due to external validation.
